# Black Ginseng Extract Counteracts Streptozotocin-Induced Diabetes in Mice

**DOI:** 10.1371/journal.pone.0146843

**Published:** 2016-01-11

**Authors:** Jun Ho Kim, Jeong Hoon Pan, Hyung Taek Cho, Young Jun Kim

**Affiliations:** Department of Food and Biotechnology, Korea University, Sejong, Republic of Korea; University of Ulster, UNITED KINGDOM

## Abstract

Black ginseng, a new type of processed ginseng that has a unique ginsenoside profile, has been shown to display potent pharmacological activities in *in vitro* and *in vivo* models. Although red ginseng is considered beneficial for the prevention of diabetes, the relationship between black ginseng and diabetes is unknown. Therefore, this study was designed to evaluate the anti-diabetic potential of black ginseng extract (BGE) in streptozotocin (STZ)-induced insulin-deficient diabetic mice, in comparison with red ginseng extract (RGE). HPLC analyses showed that BGE has a different ginsenoside composition to RGE; BGE contains Rg5 and compound k as the major ginsenosides. BGE at 200 mg/kg reduced hyperglycemia, increased the insulin/glucose ratio and improved islet architecture and β-cell function in STZ-treated mice. The inhibition of β-cell apoptosis by BGE was associated with suppression of the cytokine—induced nuclear factor–κB—mediated signaling pathway in the pancreas. Moreover, these anti-diabetic effects of BGE were more potent than those of RGE. Collectively, our data indicate that BGE, in part by suppressing cytokine—induced apoptotic signaling, protects β-cells from oxidative injury and counteracts diabetes in mice.

## Introduction

Ginseng (*Panax ginseng* Meyer) has been widely used as a folk and conventional medicine for the prevention and/or treatment of many diseases for a long time. The fundamental compounds behind ginseng’s multiple pharmacological activities are ginsenosides [1]. In Asia, ginseng is air-dried into white ginseng or steamed at 90–100°C for 2–3 h to produce red ginseng. It has been reported that red ginseng is more pharmacologically effective than white ginseng, which may result from the heat transformation and deglycosylation of ginsenosides occurring during the steaming process [2,3].

Black ginseng, a new type of processed ginseng, is produced from white ginseng after nine cycles of steaming and drying. During the steaming process for black ginseng, ginsenosides transform into low polarity constituents through hydrolysis, isomerization and dehydration at C-3, C-6 or C-20 [4]. Black ginseng has been shown to display protective effects against obesity [5], breast cancer [6], cognitive impairment [7], and fetal alcohol syndrome [8] in animals and cell culture models. Moreover, recent studies have demonstrated that black ginseng exhibits more potent antioxidant and cholinesterase inhibitory activity than white or red ginseng [9–11]. Since ginseng, particularly red ginseng, has drawn significant scientific attention for its various biological activities, these findings highlight black ginseng as a novel promising option for health promotion.

Red ginseng has been considered beneficial as a dietary supplement for the treatment of hyperglycemia and diabetes. Indeed, supplementation with red ginseng or red ginseng extract (RGE) has been shown to improve type 1 and type 2 diabetic conditions in both animals and humans [12–14]. It is interesting that fermented red ginseng, with a different ginsenoside profile, exhibited a strong anti-diabetic effects in streptozotocin (STZ)-induced diabetic rats [15] and type 2 diabetes patients [16]. However, there is a lack of data on the efficacy of black ginseng in preventing diabetes mellitus. Thus, in this study, we investigated the protective effects of black ginseng extract (BGE), in comparison with those of RGE, against STZ-induced pancreatic β-cell failure in mice.

## Materials and Methods

### Sample preparations

Powders of red ginseng and black ginseng that were made from 6-year-old *P*. *ginseng* were supplied by the International Ginseng & Herb Research Institute (Chungnam, Korea). The powders were extracted with 10 volumes of 70% ethanol at 70°C for 12 h. The extracts were filtered, concentrated under reduced pressure and then freeze-dried. Prepared samples were stored at 4°C until future use.

### Ginsenoside analysis

An HPLC system equipped with a quaternary pump system (1260 Infinity, Agilent, CA, USA), UV detector and C-18 column (4.6 × 7.5 mm, IMtakt Corp., Kyoto, Japan) was used for ginsenoside analysis. Gradient elution was employed using 10% acetonitrile (solvent A) and 90% acetonitrile (solvent B) at A:B ratios of 90:10, 79:21, 78:22, 77:23, 76:24, 63:37, 55:45, 54:46, 52:48, and 89:11, with run times of 0, 22, 25, 36, 41, 53, 61, 66, 73, and 77 min, respectively. The flow rate was maintained at 1.3 mL/min and the detection wavelength was 203 nm.

### Animal study

**Fig 1:**
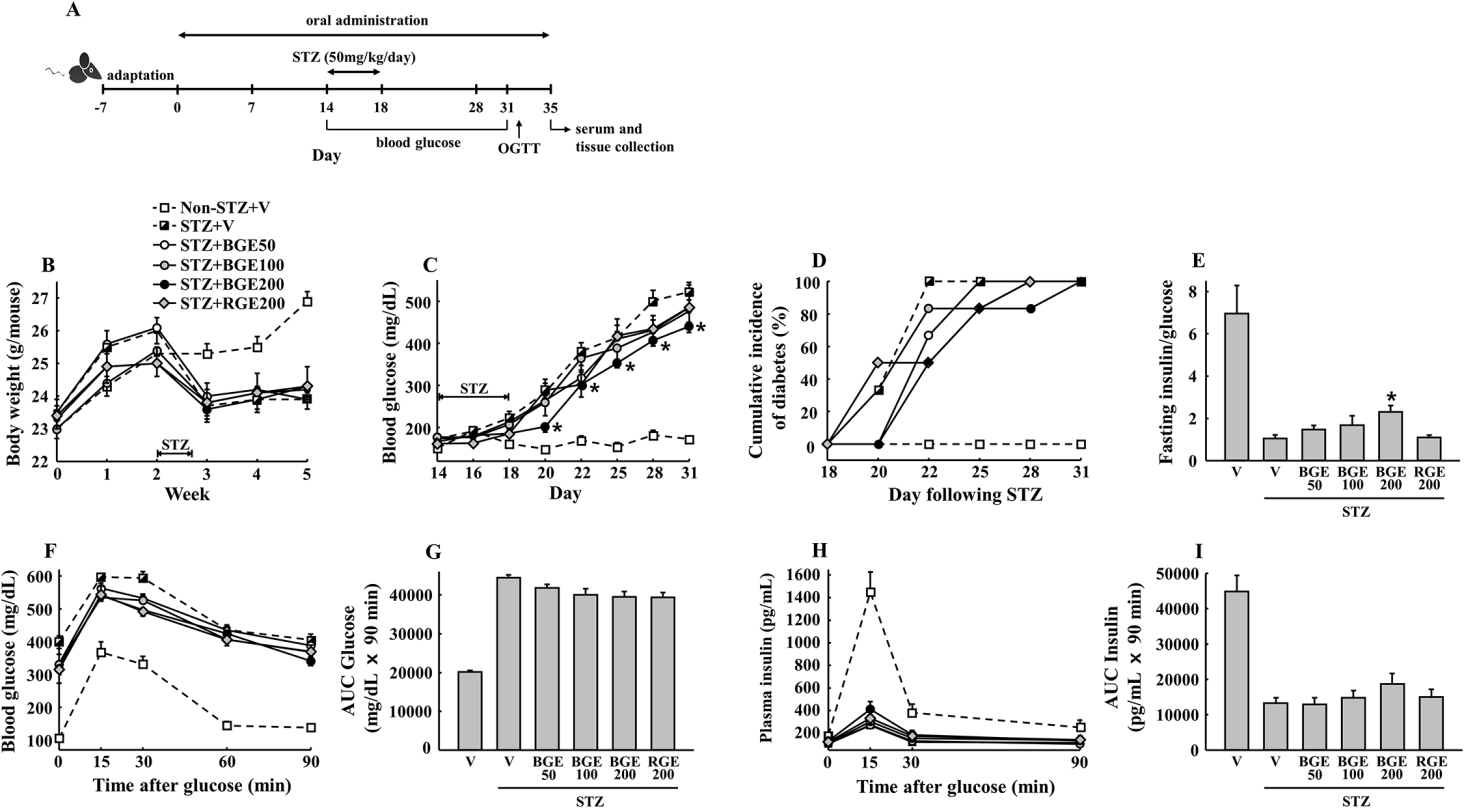
Physiological changes of glucose homeostasis. Mice received the indicated sample treatments for 5 weeks. Diabetes was induced by multiple (for 5 consecutive days) low-dose (50 mg/kg) intraperitoneal injections of STZ. (A) Time line of the study. (B) Body weights and (C) fed blood glucose during the experimental period. (D) Cumulative incidence of diabetes was calculated as a percentage of hyperglycemic mice (glucose level ≥ 300 mg/dL) at each time point. (E) The ratio of fasting insulin (pg/mL) and glucose (mg/dL) at day 32 was used as an index of insulin deficiency in mice. (F) Glucose concentrations during an oral glucose tolerance test (OGTT), (G) area under the curve (AUC) for glucose, (H) insulin concentrations during OGTT and (I) AUC for insulin. V, vehicle; BGE, black ginseng extract; RGE, red ginseng extract. Values represent means ± SEM (n = 6). *P < 0.05.

All animal work was carried out in strict accordance with the recommendations in the Guide for the Care and Use of Laboratory Animals of the National Institutes of Health. The protocol was approved by the Committee on the Ethics of Animal Experiments of Korea University (*Permit Number*: *KUIACUC-2015-127*). A timeline of the study is shown in Fig 1A. Male 8-week-old C57BL/6J mice (Central Lab. Animal Inc., Seoul, Korea) were housed with a 12-h light-dark cycle. After an adaptation period of 1 week, mice were divided into 6 treatment groups as follows: 1) non-STZ + vehicle, 2) STZ + vehicle, 3) STZ + BGE50, 4) STZ + BGE100, 5) STZ + BGE200 and 6) STZ + RGE200. All compounds were administered by gavage using an esophageal cannula once daily for 5 weeks with vehicle (normal saline), BGE 50, 100 or 200 mg/kg or RGE 200 mg/kg in saline. Induction of diabetes was initiated after 2 weeks of oral administration: mice were treated intraperitoneally for 5 consecutive days with either 50 mg/kg STZ (dissolved in 50 mM citrate buffer, pH 4.5) or citrate buffer alone. All mice received a normal chow diet with water *ad libitum* during the experimental period. At the end of the study, the mice were euthanized by an overdose of avertin, and blood was collected by cardiac puncture.

### Glucose and insulin measurements

Random-fed blood glucose was measured from blood obtained from the tail vein by using a OneTouch Ultra 2 glucose meter (LifeScan, Inc., Milpitas, CA, USA). Hyperglycemia was defined as a non-fasting blood glucose level ≥ 300 mg/dL. The cumulative incidence of diabetes was calculated as the percentage of hyperglycemic mice at each time point. An oral glucose tolerance test (OGTT) was performed at 2 weeks after STZ treatment. Mice were fasted overnight (16 h), and a glucose load (2 g/kg) was administered orally. Blood glucose and plasma insulin levels were measured from the tail vein at the indicated time points after administration of glucose. The area-under the curve (AUC) for glucose and insulin was calculated for each group of animals during the OGTT. Following overnight fasting, plasma was separated and used to determine the insulin levels by using an ELISA kit (Millipore Co., Billerica, MA, USA).

The pancreas was isolated, homogenized in acidified ethanol, extracted overnight at 4°C, and centrifuged. The insulin content of the supernatant was determined using an ELISA kit (Millipore) and expressed as ng/mg pancreas.

### Biochemical assays

Serum level of nitrite was measured using a colorimetric assay kit (Promega, WI, USA) that involves the Griess reaction. Serum level of thiobarbituric acid reactive species (TBARS) was determined by a colorimetric method as previously described [17]. The activities of superoxide dismutase (SOD) and glutathione peroxidase (GSH-Px) in the serum were determined using commercial assay kits (BioAssay Systems, CA, USA) as specified by the manufacturer. Absorbance was measured using a microplate spectrophotometer (BioRad, CA, USA).

### Histology and immunostaining

A portion of the pancreatic tail was fixed in 10% buffered formalin, embedded in paraffin, sectioned and stained with Hematoxylin and Eosin (H&E). For fluorescent microscopy imaging of insulin and glucagon, paraffin sections (2 μm) were put onto microscope slides, deparaffinized in xylene, and hydrated through graded ethanol to distilled water. Tissue sections were permeabilized in PBS with 0.1% Triton X-100 for 10 min. Blocking was performed using PBS with 5% normal goat serum for 1 h at room temperature, followed by incubation with rabbit anti-insulin antibody (Cell Signaling Technology, Danvers, MA, USA) and mouse anti-glucagon antibody (Abcam, Cambridge, UK) diluted 1:100 at 4°C overnight. Three consecutive washes with PBS for 5 min each were followed by sequential incubation with Alexa Fluor 595 and 488 goat anti-rabbit and anti-mouse IgG secondary antibodies (1:200) at room temperature for 1 h. The slides were washed three times with PBS and mounted using an anti-fade mounting medium containing 4',6-diamidino-2-phenylindole (DAPI) (Vector Laboratories, Burlingame, CA, USA). Images were captured under a fluorescence microscope (Carl Zeiss AG; Oberkochen, Germany). Islet area was determined from at least five different islets per pancreas section stained with H&E using Image J software (National Institute of Health, NIH Version v1.32j).

### Measurement of apoptosis

Terminal deoxynucleotidyl transferase-mediated dUTP nick-end labeling (TUNEL) assay was performed using *In situ cell death detection kit POD* (Roche Diagnostics, Basel, Switzerland) following the manufacturer’s instructions. Nuclei of the tissues were counterstained with DAPI and visualized as blue under a fluorescence microscope. The fluorescein isothiocyanate (FITC)-labeled tissue and cells undergoing apoptosis were recognized by their green fluorescent nuclei. Cleaved caspase-3 (the active form) levels in islet tissue sections were measured by immunofluorescence. The percentage of apoptosis was calculated by dividing the number of TUNEL-positive cells or cleaved caspase-3 positive area by the total number or area of cells stained nuclei. At least five different islets per pancreas section and four mice per condition were counted.

### Western blot analysis

Antibodies were obtained from the following sources: caspase-3, cleaved caspase-3, NF-κB p65, p-NF-κB p65, and TNF-α (Cell Signaling Technology); IFN-γ, IL-1β and GLUT2 (abcam); iNOS (Santa Cruz Biotechnology, Dallas, TX, USA). Conventional immunoblotting procedures were used to detect the target proteins. Pancreata were collected in order to extract protein using a RIPA buffer (Cell Signaling Technology). Tissue lysates were then cleared by centrifugation at 15,000 *g* for 20 min. Total protein concentration was determined by Bradford assay. Equal amounts of protein were separated on 12% SDS/PAGE and the proteins were transferred to polyvinylidene difluoride membranes. The membranes were blocked for 30 min in a PBS solution containing 3% BSA and 0.1% Tween-20 and then probed with the primary antibody overnight in a PBS solution containing 0.5% BSA and 0.1% Tween-20. After washing, the membranes were incubated for 1 h with horseradish peroxidase-linked secondary antibodies (Sigma-Aldrich, St. Louis, MO, USA) in a PBS solution containing 0.5% BSA and 0.1% Tween-20. Finally, after three 10-min washes in 0.1% PBS/Tween-20, proteins were visualized using ImageQuant LAS 4000 (General Electric, Pittsburgh, PA, USA).

### RT-PCR analysis

Pancreatic tissue was homogenized in 1 mL of TRIzol reagent, and then total RNA was isolated according to the TRIzol protocol. Total RNA was reverse transcribed to cDNA using a High Capacity cDNA Reverse Transcription kit (Applied Biosystems, Foster, CA) as per the manufacturer’s protocol. cDNA was used as a template for the relative quantitation of the selected target genes with predesigned TaqMan gene expression assay kits. Each 20 μL reaction contained 100 ng cDNA, 2× TaqMan Gene Expression Mastermix, forward and reverse primers and TaqMan probe. All reactions were carried out in triplicate with an ABI 7500 system (Applied Biosystems) using the following conditions: 50°C for 2 min and 95°C for 10 min, followed by 40 cycles of 95°C for 15 s and 60°C for 1 min. Results were normalized to GAPDH as an internal standard, and the relative quantity of each gene was presented in terms of 2^-ΔΔCt^, calculated using the ΔCt and ΔΔCt values.

### Statistical analysis

Data were analyzed by one-way ANOVA using the SAS software for Windows release 9.2 (SAS Institute Inc., Cary, NC) on the W32_VSHOME platform. One-way ANOVA with repeated measures was performed to assess mean differences between groups for body weight and blood glucose over time. The least squares means option using a Tukey-Kramer adjustment was used for multiple comparisons among the experimental groups. Data are shown as the mean ± SE. *P* values of < 0.05 are reported as statistically significant.

## Results

### Rg5 and compound k are major ginsenoside in BGE but not in RGE

HPLC analyses showed the different ginsenoside profiles between RGE and BGE (Table 1). Major ginsenosides in RGE (> 3 mg/g) were Rb1, Re and Rg1, while those in BGE were Rg5 and compound k. The chemical structures of these ginsenosides are shown in S1 Fig.

**Table 1:** Ginsenosides contents (mg/g) of red and black ginseng extracts.

	RGE	BGE
Rb1	8.1 ± 0.1	1.5 ± 0.1
Rb2	2.7 ± 0.1	0.6 ± 0.0
Rc	2.9 ± 0.1	0.7 ± 0.0
Rd	1.2 ± 0.1	0.5 ± 0.1
Re	3.9 ± 0.1	0.5 ± 0.1
Rf	1.1 ± 0.0	0.6 ± 0.0
Rg1	3.9 ± 0.1	0.2 ± 0.0
Rg2	0.4 ± 0.0	0.8 ± 0.0
Rg3	0.2 ± 0.0	1.7 ± 0.1
Rg5	0.4 ± 0.0	4.7 ± 0.2
Rh1	0.2 ± 0.0	0.5 ± 0.0
Rh2	0.2 ± 0.1	0.2 ± 0.0
Rk1	ND	ND
F2	ND	ND
Compound k	0.7 ± 0.1	5.6 ± 0.1

RGE, red ginseng extract; BGE, black ginseng extract; ND, not detected. Values represent means ± SEM (n = 3).

### Black ginseng extract counteracts STZ-induced hyperglycemia and islet dysfunction in mice

No significant body weight change with BGE or RGE administration was observed after STZ treatment (Fig 1B). Multiple-low-dose STZ (MLDS) treatment induced progressive hyperglycemia, with a corresponding increase in diabetes incidence (blood glucose ≥ 300 mg/dL) in mice (Fig 1C and 1D). BGE at 200 mg/kg significantly reduced the increase in blood glucose level and delayed the cumulative incidence of diabetes. However, RGE at the same concentration did not show a significant difference in blood glucose compared to the STZ vehicle group. Furthermore, BGE increased fasting insulin/glucose ratio, an index of insulin resistance [18], in a dose-dependent manner, which was notably reduced by MLDS treatment (Fig 1E). Administration with a high-dose of BGE showed an improved glucose tolerance (Fig 1F and 1G) and higher plasma insulin level in response to glucose compared to the STZ vehicle (Fig 1H and 1I), but these effects were not statistically significant.

**Fig 2:**
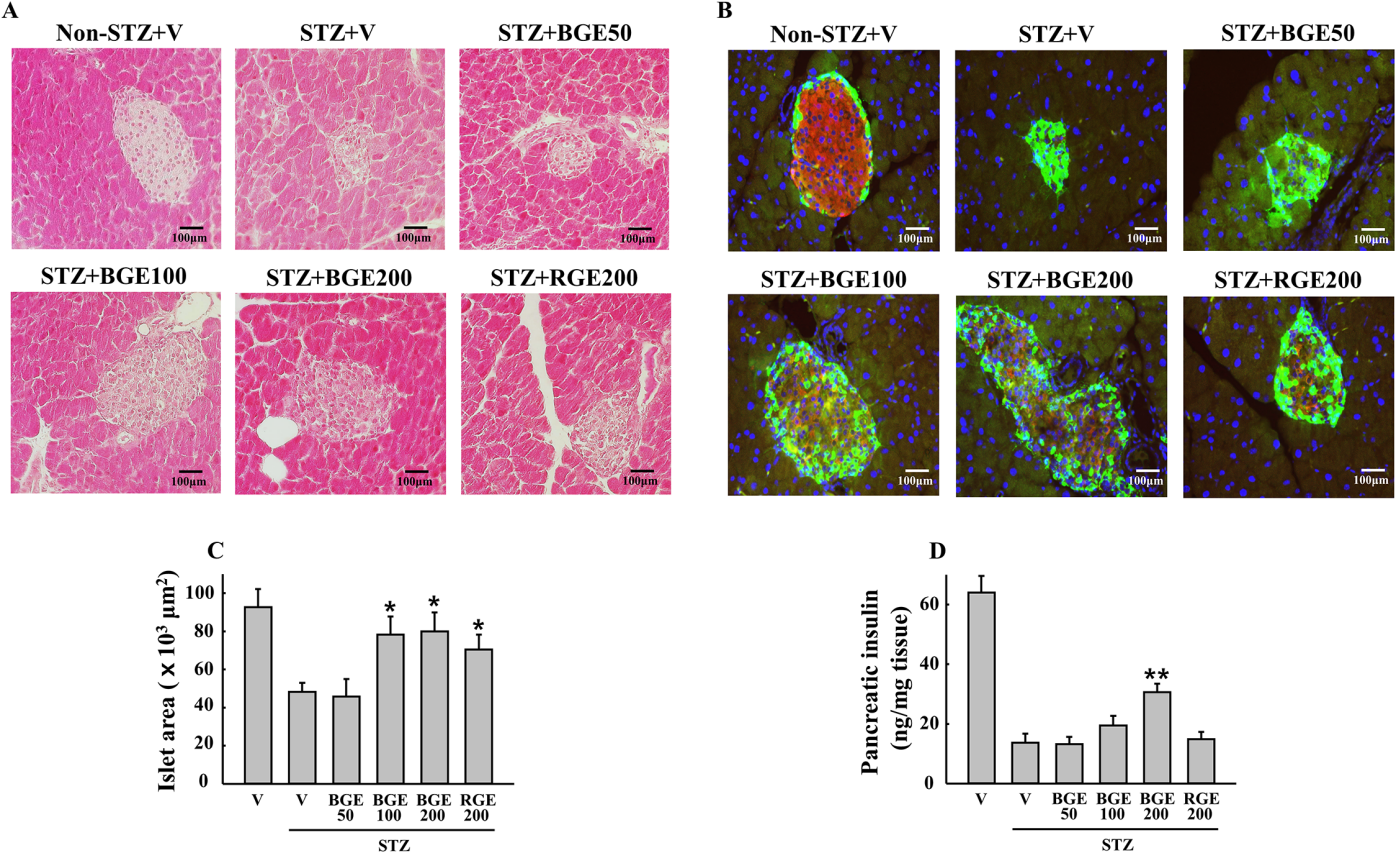
Disruption of islet architecture and function. (A) Hematoxylin and Eosin (H&E) staining and (B) immunofluorescent staining for insulin (red) and glucagon (green) were performed to analyze histopathological changes in the pancreatic islets at the end of study. (C) Islet area was determined from at least 5 different islets per pancreas stained with H&E (n = 4). (D) Pancreatic insulin concentration measured by ELISA (n = 6). V, vehicle; BGE, black ginseng extract; RGE, red ginseng extract. Values represent means ± SEM. *P < 0.05; **P < 0.01.

Consistent with the changes of blood glucose and insulin, the STZ-treated mice with a high-dose of BGE showed improved islet architecture, with insulin-producing β-cells in a central location and glucagon-producing α-cells at the periphery, compared with the STZ vehicle mice (Fig 2A and 2B). BGE and RGE at 100 or 200 mg/kg reversed the STZ-induced reduction of islet size; however, the pancreatic insulin content in STZ-treated mice significantly increased only with BGE at 200 mg/kg (Fig 2C and 2D).

### Black ginseng extract suppresses STZ-induced β-cell apoptosis in mice

**Fig 3:**
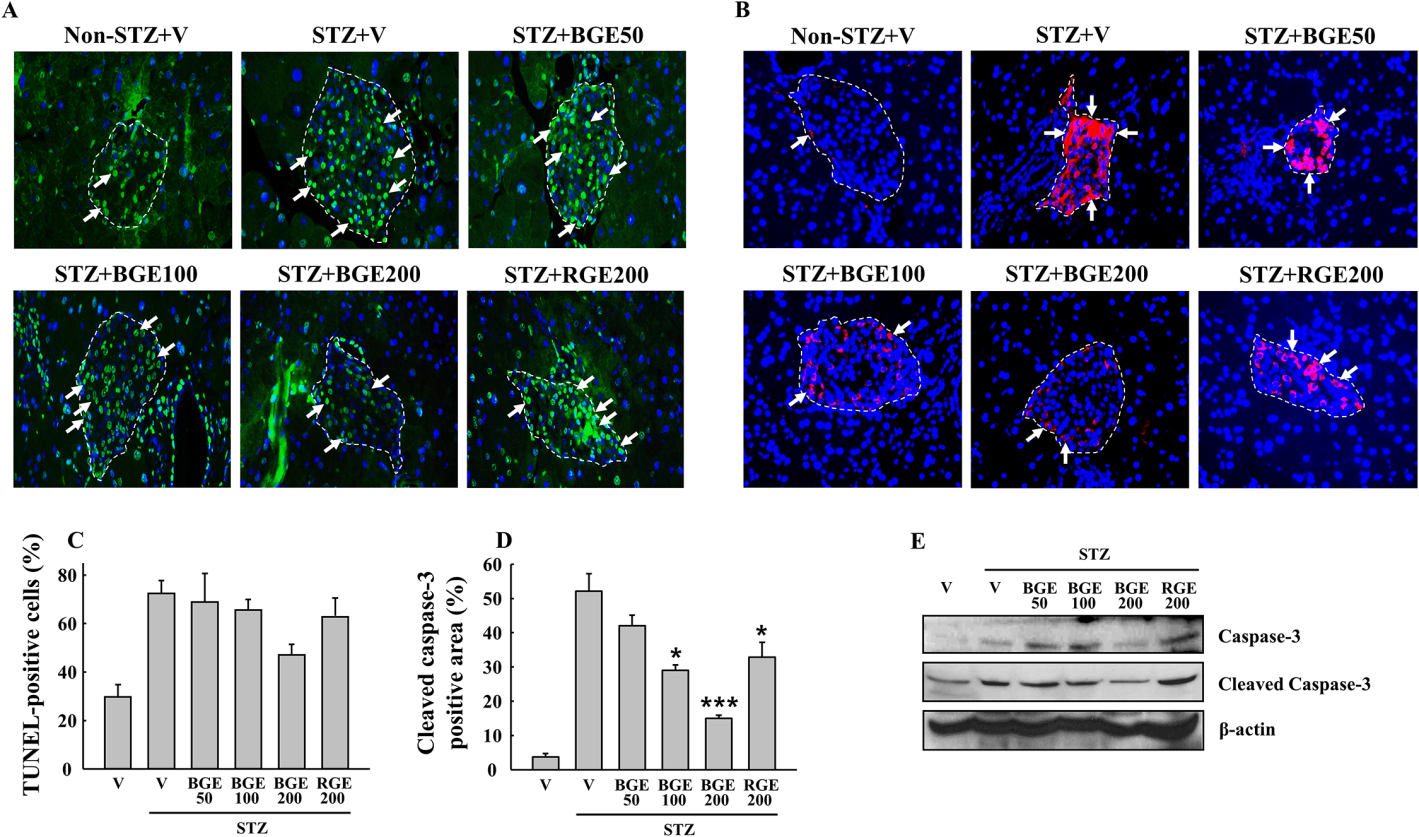
STZ-induced β-cell apoptosis. (A) TUNEL (green) and (B) active caspase (red) immunofluorescent staining were performed to measure apoptosis in pancreatic islets at the end of study. Arrows indicate cells with positive signals. At least five different islets per pancreas section were counted to calculate the percentage of (C) TUNEL-positive cells or (D) cleaved caspase-3 positive area. (E) Pancreatic protein expressions of total caspase-3 and cleaved caspase-3 were measured by western blotting. V, vehicle; BGE, black ginseng extract; RGE, red ginseng extract. Values represent means ± SEM (n = 4). *P < 0.05; ***P < 0.001.

The treatment with STZ shows a selective targeting of β-cells for apoptosis but not α-cells or extra-pancreatic parenchyma [19]. As reflected by TUNEL and active caspase staining, we observed a major increase in apoptotic β-cells in the STZ vehicle mice (Fig 3A–3D). In contrast, BGE administration at 200 mg/kg suppressed apoptosis, as shown by both TUNEL and active caspase staining, demonstrating the important role of BGE in preventing acute oxidative injury *in vivo*. Importantly, this effect of BGE was more potent than that of RGE: the effect of BGE at 100 mg/kg was comparable to that of RGE at 200 mg/kg. Western blot analyses of caspase-3 showed that BGE at 200 mg/kg suppressed the expression of both total and cleaved caspase-3 protein in the pancreas (Fig 3E).

### Black ginseng extract inhibits STZ-induced inflammatory responses in pancreas of mice

**Fig 4:**
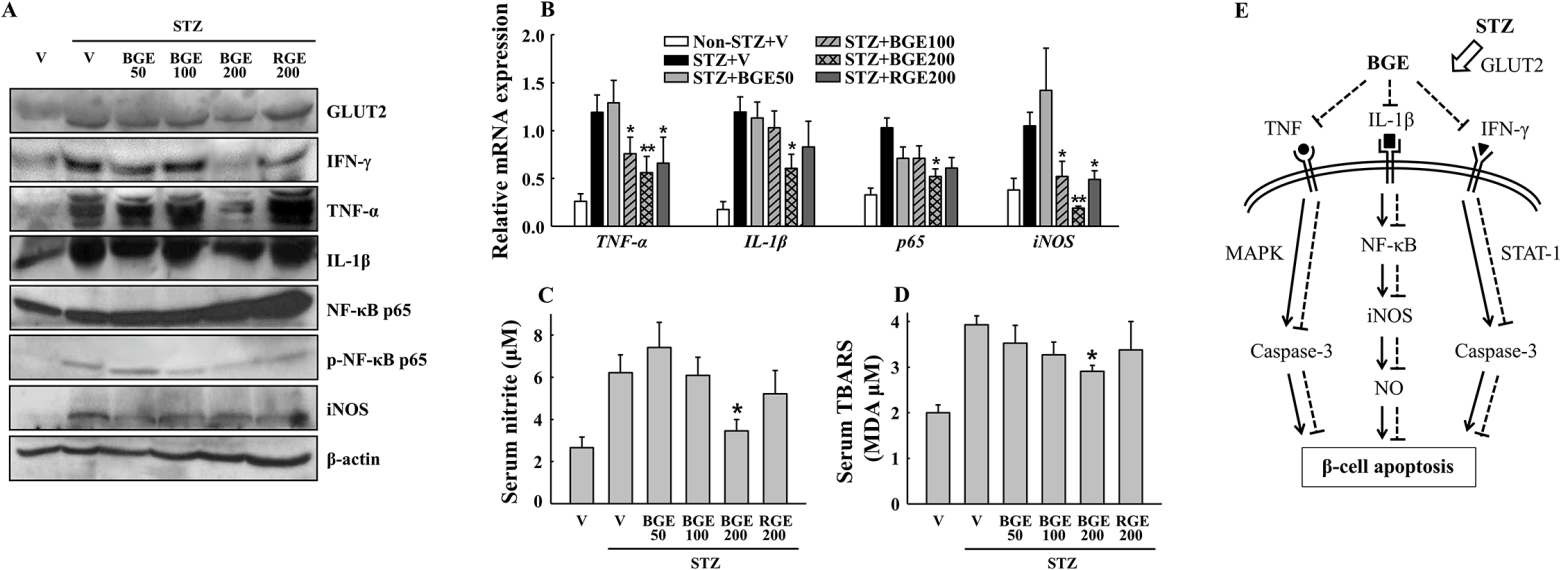
Cytokine-induced toxic signaling in β-cells. (A) Pancreatic protein expressions of glucose transporter 2 (GLUT2), interferon-γ (IFN-γ), tumor necrosis factor-α (TNF-α), interleukin-1β (IL-1β), nuclear factor-κB p65 (NF-κB p65), phospho-NF-κB p65 (p-NF-κB p65) and inducible nitric oxide synthase (iNOS) were measured by western blotting (n = 4). (B) Pancreatic mRNA expressions of *TNF-α*, *IL-1β*, *p65* and *iNOS* (n = 6). (C) Nitrite and (D) thiobarbituric acid reactive substances (TBARS) concentrations in serum (n = 6). TBARS was expressed in terms of malondialdehyde (MDA) equivalents. (E) A potential inhibitory mechanism of STZ-induced β-cell apoptosis by BGE. STZ is taken up by β-cells via GLUT2 and promotes cytokine-initiated apoptotic signaling. BGE may suppress the expressions of IFN-γ, TNF-α and IL-1β, leading to inhibition of the NF-κB pathway and caspase-3 activation, which ultimately protects the β-cells. Solid lines indicate the stimulatory actions by STZ. Dashed lines indicate the inhibitory actions by BGE. V, vehicle; BGE, black ginseng extract; RGE, red ginseng extract. Values represent means ± SEM. *P < 0.05; **P < 0.01.

To determine the underlying mechanisms involved in the suppression of STZ-induced β-cell failure by BGE, we next examined the changes in cytokine-mediated inflammatory responses in the pancreas. Our results show that the protein and/or mRNA expressions of IFN-γ, TNF-α, IL-1β, phospho-NF-κB p65 and iNOS greatly increased in STZ vehicle mice, but not in the STZ-treated mice administered 200 mg/kg BGE (Fig 4A and 4B), suggesting that BGE administration inhibits the cytokine-initiated NF‑κB pathway in the pancreas. Moreover, the serum levels of nitrite and TBARS, markers of oxidative stress, were significantly decreased by 200 mg/kg BGE in STZ-treated mice compared to corresponding levels in the vehicle treatment (Fig 4C and 4D). However, no difference was found in serum SOD and GSH-Px activities among all tested groups (data not shown). In all STZ treatment groups, there were no differences in the protein expressions of the GLUT2 transporter that allows for the apoptotic effect of STZ in β-cells [20]. A potential inhibitory mechanism of STZ-induced β-cell apoptosis by BGE is illustrated in Fig 4E.

## Discussion

This study demonstrates that administration of BGE counteracts MLDS-induced diabetes in mice, and that this effect is associated with suppression of cytokine-induced inflammatory signaling. Furthermore, BGE exhibited stronger effects on β-cell protection than RGE at the same dosage. Although recent evidence indicates that BGE, with a unique ginsenoside profile, has a more potent antioxidative and cholinergic functions than RGE [9,11], our study is the first report to focus on β-cell function in a diabetic animal model with administration of RGE and BGE.

The first finding of this study is that BGE counteracts MLDS-induced islet deterioration, β-cell apoptosis and insulin deficiency in mice in a dose-dependent manner, although overall significant effects were observed at a 200 mg/kg dose (Figs 1–3). However, in this study, we failed to observe significant improvements in MLDS-induced hyperglycemia and insulin deficiency by RGE. This is inconsistent with previous findings that showed the protective effects of RGE against MLDS- or cyclosporine-induced diabetic conditions in mice [13,21]. This discrepancy of efficacy could be explained by rapid increases of MLDS-induced hyperglycemia and the lower dose of RGE used in our study. Indeed, our data showed higher blood glucose levels (≥ 500 mg/dL) in the vehicle group after 2 weeks of MLDS treatment compared to blood glucose levels (about 350 mg/dL) after 4 weeks of MLDS treatment in the study by Hong et al. [13]. Consistent with our data, RGE at the same dose (200 mg/kg) had no effect on hyperglycemia and other diabetic parameters when mice had a high blood glucose level (≥ 600 mg/dL) with single high-dose STZ treatment [12]. Therefore, these results suggest that the anti-diabetic effects of BGE observed in our study could be more remarkable under a mild or moderate diabetic condition.

The second observation is the suppression of inflammatory signaling involved in cytokine-induced β-cell apoptosis by BGE. STZ is taken up by pancreatic β-cells via the GLUT2 transporter in the plasma membrane, and it causes β-cell death via multiple mechanisms including DNA methylation, nitric oxide (NO) production and the generation of reactive oxygen species [19]. A combination of IFN-γ, TNF-α and IL-1β promotes NO production and activates a series of caspase cysteine proteases via NF-κB- or STAT1-mediated signaling pathway, which ultimately induce β-cell apoptosis [22]. In the current study, protection of β-cell function in BGE-treated mice was accompanied by down-regulations of IFN-γ, TNF-α, IL-1β, NF-κB p65 and iNOS mRNA and/or protein expressions (Fig 4). Our data also showed reduced serum levels of nitrite and TBARS (Fig 4) and inhibition of caspase-3 activation (Fig 3) in the STZ+BGE200 group compared to the STZ vehicle. These results clearly indicate that BGE attenuates cytokine—induced NF-κB—mediated apoptosis signaling in STZ-treated mice. However, we observed no effect on GLUT2 expression (Fig 4), ruling out the possibility that BGE inhibits apoptotic signaling by blocking STZ entry into β-cells via GLUT2.

HPLC analyses showed that Rg5 and compound k are the major ginsenoside compounds in BGE, but are present only in negligible amounts in RGE (Table 1). These data are similar to those of other studies that have reported the ginsenoside compositions in white, red and black ginsengs, although there are some differences according to the processing and analytical conditions [4,9]. Ginsenoside Rg5 and compound k have both been shown to display anti-inflammatory effects by suppression of proinflammatory cytokine production and NF-κB signaling in both *in vitro* and *in vivo* models [23–26]. It was recently reported that oral administration of Rg5 attenuated neuroinflammatory responses in STZ-treated rats [27]. In addition, in a diabetic mouse model induced by a high-fat diet with STZ, compound k showed greater hypoglycemic and insulin-sensitizing effects than protopanaxadiol-type ginsenosides (e.g. Rb1, Rb2, Rc, Rd, Rg3 and Rh2), which are metabolized by intestinal bacteria to compound k [28]. Along with these previous reports, our current data suggest that Rg5 and compound k might be the ginsenosides responsible for the counteracting effects of BGE on STZ-induced inflammatory responses and β-cell failure.

Since black ginseng, a new type of processed ginseng, has been shown to exhibit potent pharmacological activities, its potential health concerns are important issues to address. Recently, Jin et al. [9] reported the detection of carcinogenic benzo(a)pyrene in BGE (0.17 μg/kg) at less than the maximum level that the Korea Food and Drug Administration established for benzo(a)pyrene in BGE (4.0 μg/kg). In the current study, however, benzo(a)pyrene was not detectable in either RGE or BGE (data not shown). Nevertheless, others have reported that the benzo(a)pyrene content in black ginseng could be varied depending on the steaming time and temperature [29,30]. Thus, further investigations are needed to standardize the optimal conditions for the production of functional ginsenoside-enhanced black ginseng, without generation of carcinogenic benzo(a)pyrene.

Taken together, our results demonstrate that BGE containing Rg5 and compound k in high proportions counteracts STZ-induced diabetes in mice with more potent effects than RGE. This protection occurs by down-regulating proinflammatory cytokine expression and blocking NF-κB activation in the pancreas. These observations provide evidence for a novel functional role of black ginseng and warrant further clinical evaluation for the prevention of insulin-dependent diabetes.

## Supporting Information

S1 Fig The chemical structures of the major ginsenosides in RGE (Rb1, Re and Rg1) and BGE (Rg5 and compound k). (TIF)
