# Supplementary figures and images for: Black Ginseng Extract Counteracts Streptozotocin-Induced Diabetes in Mice
Source: PLoS One. 2016 Jan 11;11(1):e0146843. doi: 10.1371/journal.pone.0146843 (PMC4709037; doi:10.1371/journal.pone.0146843)

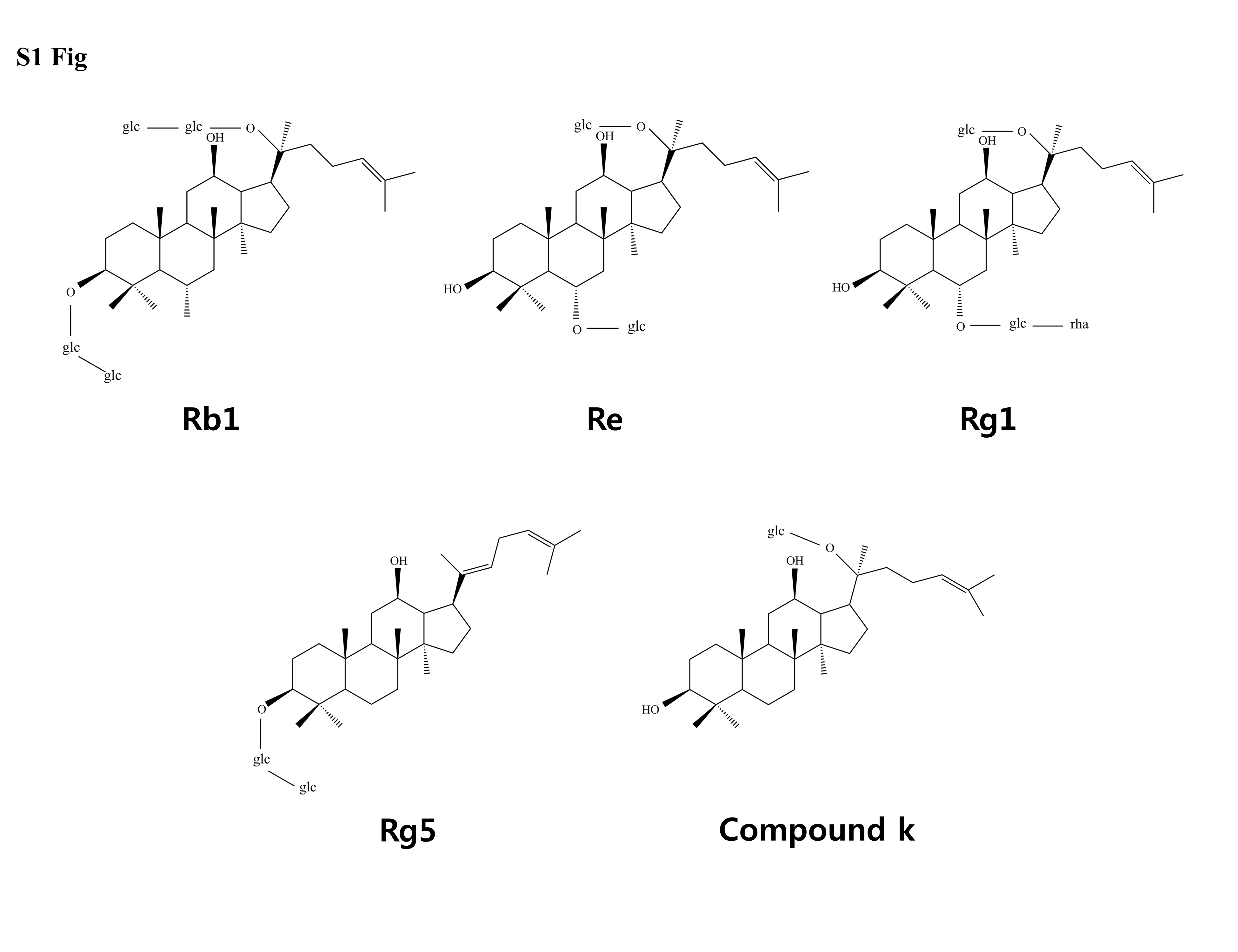

Supplement: S1 Fig — (TIF) [file pone.0146843.s001.tif]
